# Supplementary material for: Collaborative intelligence in AI: Evaluating the performance of a council of AIs on the USMLE
Source: PLOS Digit Health. 2025 Oct 9;4(10):e0000787. doi: 10.1371/journal.pdig.0000787 (PMC12510544; doi:10.1371/journal.pdig.0000787)
Supplement: S2 File — (ZIP) [file pdig.0000787.s002.zip › S2 Data Raw data files containing unprocessed question inputs and LLM outputs including transcripts of deliberations/STEP3_Question-25-1_Type-MC-NJ.docx]

**Facilitator** **[Round 1]:** A 23-year-old man comes to the office because of a 7-day history of fever, crampy abdominal pain, and diarrhea. He says the abdominal pain worsens with oral intake. He has been having 8 to 10 episodes of diarrhea daily. He has not traveled recently and has had no sick contacts. Medical history is unremarkable and he takes no medications. He does not smoke cigarettes, drink alcoholic beverages, or use illicit drugs. He is not sexually active. Vital signs are temperature 38.3°C (101.0°F), pulse 118/min, respirations 18/min, and blood pressure 108/58 mm Hg. Bowel sounds are hyperactive. The abdomen is diffusely tender to palpation; no masses are palpated. Neither the liver edge nor the spleen can be palpated. Digital rectal examination shows grossly bloody stool. A stool culture is obtained, and empiric pharmacotherapy is initiated. Which of the following microorganisms is the most likely cause of this patient's condition? (A) Campylobacter jejuni (B) Candida albicans (C) Enterovirus (D) Giardia lamblia

**AI_D:** (1) RESTATING THE QUESTION: We have a young male presenting with a week-long history of fever, diarrhea, and abdominal pain that worsens with food consumption. The symptoms and findings, including hyperactive bowel sounds, unremarkable past medical history, absence of recent travel or sick contacts, tender abdomen, and bloody stool, suggest an ongoing infection or inflammation in the gastrointestinal tract. The aim here is to identify the most likely infectious microorganism causing his condition, among the choices provided: Campylobacter jejuni, Candida albicans, Enterovirus, or Giardia lamblia. (2) PLANNING: Consideration is given to each potential causative organism. Campylobacter jejuni is often associated with undercooked poultry consumption and can cause bloody diarrhea. Candida albicans is usually an opportunistic fungus and usually affects immunocompromised individuals. Enterovirus, on the other hand, can lead to a variety of symptoms but rarely causes bloody stool. Giardia lamblia is a common cause of traveler's diarrhea, but our patient hasn't traveled recently. (3) CLINICAL REASONING: Drawing upon knowledge from microbiology and internal medicine, among the provided choices, Campylobacter jejuni is known to cause a clinical presentation highly akin to what's seen in the patient. Symptomatically, Campylobacter jejuni infection matches with what the patient is experiencing, as it can lead to high fever, abdominal pain and, importantly, bloody stools. This bacteria is one of the most common causes of infectious diarrhea worldwide. The other choices do not align as closely. Candida infection in the gut usually follows a course of broad-spectrum antibiotics or in immunocompromised individuals. Enterovirus infection typically presents with different symptoms including cough, rash, mouth sores, and rarely presents with grossly bloody stools. Giardia is not typically associated with the severe symptoms seen in this patient and would have been a more likely consideration if there had been a recent travel history, which is absent in this case. (4) CONCLUDING: With the above clinical reasoning, the symptoms of bloody stools, fever, and abdominal pain, the absence of recent travel, lack of immunosuppression, and the severity of symptoms suggest a bacterial cause in line with what we see in Campylobacter jejuni infections. (5) RESPONSE: Therefore, Campylobacter jejuni (choice A) is the most plausible microorganism causing this patient's condition.

**AI_A:** RESTATING THE QUESTION: We are asked to identify the most likely pathogen causing the symptoms experienced by a 23-year-old man. These symptoms include, but are not limited to, a week-long history of fever, crampy abdominal pain that worsens upon oral intake, and frequent, bloody diarrhea. Additionally, he doesn't have recent travel history or exposure to sick contacts, and his medical and social history do not suggest any related risk factors. PLANNING: A crucial assumption is that the patient's symptoms are indicative of a bacterial, viral, or protozoan infection given the choice of microorganisms presented. This assumption can be questioned as other causes of similar symptoms such as physiological and anatomical abnormalities, allergic reactions, or autoimmune diseases are not completely ruled out. However, due to the presence of fever and the nature of his symptoms, an infectious cause appears most plausible. On that basis, comparing the pathogenesis and usual clinical presentation of the four suggested microorganisms (Campylobacter jejuni, Candida albicans, Enterovirus, Giardia lamblia) should help us determine the most likely culprit. CLINICAL REASONING: Campylobacter jejuni is a bacterium known to cause gastroenteritis with fever and bloody stools, which generally starts 2 to 5 days after exposure. It is transmitted through contaminated water or undercooked meat, posing an occupational risk to those in the food-handling industry or related fields, but our patient didn't have such exposure. Candida albicans is typically observed in immunosuppressed individuals or patients on prolonged antibiotics, neither of which applies to our patient. An Enterovirus infection commonly present with a mix of respiratory and gastrointestinal symptoms in children rather than isolated gastrointestinal symptoms in adults. Giardia lamblia, a protozoan, causes diarrhea, but it generally doesn't cause bloody stool or a fever. Also, it's typically associated with recent travel or ingestion of contaminated water, which our patient hasn't reported. CONCLUDING: After evaluating the characteristic presentations associated with each of these pathogens, Campylobacter jejuni seems the most likely to be responsible for our patient's symptoms given its clinical presentation and lack of specific risk factors or clinical history that would suggest the other pathogens. RESPONSE: (A) Campylobacter jejuni, is the most likely cause of this patient's condition.

**AI_B:** RESTATING THE QUESTION: A young man has come to the clinic with a 7-day history of fever, abdominal cramps, and diarrhea. He also experiences worsened abdominal pain upon ingesting food. He gets diarrhea 8 to 10 times daily. He hasn't traveled or been around sick people recently, and he has a clean medical history; he has no drug or alcohol habits, prescription or otherwise. No sexual activity also rules out sexually transmitted diseases. His vital signs indicate fever, tachycardia, but normal respiratory rate and blood pressure. Physical examination reveals profoundly active bowel sounds, diffuse abdominal tenderness but no palpable masses, liver or spleen. Blood in the stool is evident on performing a digital rectal examination, and this prompts a stool culture; empiric treatment has also begun. The question is concerning the most likely microbiological agent causing these symptoms. PLANNING: The differential diagnosis for a young patient with this sort of symptomatology can include various bacterial, viral, parasitic, and fungal pathogens. Our patient's presentation - fever, abdominal pain, hyperactive bowel sounds, bloody diarrhea, and lack of epidemiological clues such as recent travel or sick contacts - points towards an infection more likely caused by an invasive organism capable of causing inflammation and bleeding within the bowel tract. Noteworthily, cases of Campylobacter jejuni often originate from eating raw or undercooked poultry, or from contaminated milk or water, and frequently present with symptoms similar to those displayed by our patient. On the other hand, Candida albicans is typically a cause of thrush and not typically associated with diarrheal disease unless the patient is immunocompromised. The same goes for Enterovirus which typically causes symptoms such as fever, rash, and respiratory, but not usually bloody diarrhea. Giardia lamblia often leads to chronic diarrhoea with a fatty, bad-smelling stool but not usually associated with bloody diarrhea. CLINICAL REASONING: Considering the clinical signs of fever, abdominal pain, and bloody diarrhea together with the lack of significant epidemiological hints suggest that we are dealing with an invasive organism that is causing inflammation and bleeding within the bowel tract. Campylobacter jejuni fits this clinical picture better than the other options: it is a common cause of bacterial enteritis worldwide, and can cause bloody diarrhea. CONCLUDING: Going through the clinical reasoning process, we start with the characteristic features of the case, analyze the possible pathogens stated in the question, check their pathogenesis and typical clinical presentations, and then decide which of them fits our case the best. RESPONSE: The most probable pathogen causing the 23-year-old man's diarrheal illness is Campylobacter jejuni (option A), taking into account his symptoms and the characteristics of the said microorganism in human disease.

**AI_E:** RESTATING THE QUESTION: You are inquiring about a 23-year-old man with a seven-day history of fever, abdominal pain, and diarrhea. The abdominal pain intensifies during the ingestion of food. Daily, the patient experiences between 8 to 10 episodes of diarrhea. His medical history is relatively routine and he does not take any medication or partake in any smoking, drinking or drug use activities. He is not sexually active. Vital findings illustrate that his body temperature is 38.3°C (101.0°F), pulse rate is 118/min, respiratory rate is 18/min, and blood pressure is 108/58 mm Hg. His bowel sounds are in a hyperactive state, and his abdomen is tender all over, however, no masses could be felt during the palpation. Neither the edge of the liver nor the spleen could be palpated. A rectal examination revealed the presence of blood in the stool. Consequently, a stool culture was taken and empiric medication was started. Among the given options, you want to know which microorganism was the most likely culprit for this patient's condition. PLANNING: In this case, we are dealing with a young man who is showing signs of gastroenteritis, as indicated by his symptoms of abdominal pain, fever, and bloody diarrhea. We need to understand that gastroenteritis can be caused by various pathogens including bacteria, viruses, and parasites. Each pathogen would behave differently and could potentially be associated with other distinct symptoms or epidemiological clues. However, in this particular case, the lack of traveling or sick contact history and the presence of bloody diarrhea are key features. We can rule out most of the listed options: Candida albicans (fungal) is unlikely as the patient has no history of immune suppression. Giardia lamblia, largely associated with waterborne outbreaks primarily in travelers, makes it less likely in a non-traveler patient. Enterovirus, while can cause gastroenteritis, is less likely to cause bloody diarrhea. CLINICAL REASONING: Bloody diarrhea, also known as dysentery, is often associated with invasive bacterial pathogens. These are typically Campylobacter, Shigella, Salmonella, E. coli, etc. Despite the absence of noted history of consumption of uncooked food or recent travel, the current patient's signs and symptoms indicate an invasive enteritis condition. This pattern of invasive enteritis is classic for Campylobacter jejuni, which can commonly lead to bloody diarrhea and systemic symptoms of fever and malaise. CONCLUDING: After the careful analysis of the provided information and collecting the important findings, we can identify that the pathogen responsible for the symptoms of the patient seems to be a bacterium that causes invasive diarrhea. Out of the options provided, "Campylobacter jejuni" fits best with this description. RESPONSE: Therefore, the most probable microorganism causing this patient's condition is Campylobacter jejuni.

**AI_C:** (1) RESTATING THE QUESTION: This is a case of a 23-year-old man who has shown up to the clinic with a seven-day history of fever, severe abdominal pain that exacerbates when he consumes food, and a high number of diarrheal episodes per day. He also has not been in contact with anyone who is sick, has not been traveling lately, and has no substantive medical history. His vitals reveal a slightly high temperature, an elevated pulse, normal respiration and slightly low blood pressure. Upon physical examination, his bowel sounds were found to be hyperactive and his abdomen seemed to be diffusely tender, though no masses were detected. Rectal examination resulted in the discovery of bloody stool. Following the necessary stool sample testing, the patient was started on an empirical treatment regimen. The task for this discussion is to hypothesize on the most probable microorganism responsible for this debilitating state of the patient. (2) PLANNING: From the symptoms presented and physical examination data, we can assume this patient is suffering from a gastroenteric condition. The key assumptions here are that it is either bacterial, fungal, viral or parasitic gastroenteritis. Each assumption can be challenged as follows: (A) Campylobacter jejuni, a bacterial infection, which could cause bloody diarrhea, however, this is often associated with raw or undercooked poultry and unpasteurized dairy, and recent travel; (B) Candida albicans, a species of yeast, might cause diarrhea, but is more common after antibiotic use or in patients with impairment of the immune system; (C) Enterovirus, a type of virus, may cause fever, abdominal pain and diarrhea, but bloody diarrhea is not a common symptom and it mostly affects children; (D) Giardia lamblia, a protozoan parasite, would cause diarrhea and abdominal cramps. But bloody diarrhea is rarely observed in giardiasis and it is commonly linked with contaminated water or food, often with a travel history. (3) CLINICAL REASONING: Through a process of exclusion, let's consider each options. (A) C.jejuni, a common cause of bacterial gastroenteritis, can cause Bloody diarrhea. However the patient had no sick contacts or ran into food safety issue recently. (B) C. albicans is usually linked with disruption of the normal gut microbiota by antibiotic treatment or immune impairment like HIV infection, also patient has no yeast infection-related symptoms and there has been no significant medical history. (C) Enteroviruses typically are highly infectious and associated with outbreaks, moreover, grossly bloody diarrhea is not typically seen in enteroviral infection. (D) Giardiasis would mostly result in greasy, foul-smelling stools without blood. Also, giardiasis is commonly associated with recent travel, but the patient has not traveled recently. Based on these, the lesser likely option would be Campylobacter jejuni. (4) CONCLUDING: Weighing the clinical presentation and the given options, though the patient did not have a food safety issue or sick contact, Campylobacter jejuni must be considered as the probably microorganism causing gastroenteritis in this young man because it is capable of causing symptoms of fever, crampy abdominal pain, and bloody diarrhea, which are all exhibited by this patient. (5) RESPONSE: Therefore, the most likely microorganism causing this patient's condition is Campylobacter jejuni.

**Facilitator** The consensus among the AI responses is that the most likely pathogen causing the symptoms experienced by the 23-year-old man is Campylobacter jejuni. This conclusion is based on the patient's symptoms of fever, crampy abdominal pain, and bloody diarrhea, which are indicative of an infectious cause, most likely bacterial. Campylobacter jejuni is known to cause gastroenteritis with fever and bloody stools, aligning with the patient's symptoms. The other options, Candida albicans, Enterovirus, and Giardia lamblia, were considered less likely due to the patient's lack of specific risk factors or clinical history that would suggest these pathogens. Candida albicans is typically observed in immunosuppressed individuals or patients on prolonged antibiotics, neither of which applies to our patient. An Enterovirus infection commonly presents with a mix of respiratory and gastrointestinal symptoms in children rather than isolated gastrointestinal symptoms in adults. Giardia lamblia, a protozoan, causes diarrhea, but it generally doesn't cause bloody stool or a fever and is typically associated with recent travel or ingestion of contaminated water, which our patient hasn't reported. Therefore, Campylobacter jejuni is the most plausible microorganism causing this patient's condition.
